# Reply to Fusco et al. Is Medication-Related Osteonecrosis of the Jaws (MRONJ) Associated to Cyclin-Dependent Kinase (CDK) 4/6 Inhibitors? A Word of Cautiousness. Comment on “Marcianò et al. Medication-Related Osteonecrosis of the Jaws and CDK4/6 Inhibitors: A Recent Association. *Int. J. Environ. Res. Public Health* 2020, *17*, 9509”

**DOI:** 10.3390/ijerph181910145

**Published:** 2021-09-27

**Authors:** Antonia Marcianò, Gian Marco Guzzo, Matteo Peditto, Antonio Picone, Giacomo Oteri

**Affiliations:** 1 Department of Clinical and Experimental Medicine, University of Messina, 98122 Messina, Italy; 2 Humanitas Istituto Clinico Catanese, Department of Medical Oncology, 95045 Misterbianco, Italy; antonio.picone@ccocatania.it; 3 Department of Biomedical and Dental Sciences and Morphofunctional Imaging, University of Messina, 98122 Messina, Italy; gianmarco.guzzo@libero.it (G.M.G.); mpeditto@unime.it (M.P.); giacomo.oteri@unime.it (G.O.)

We read the letter from Dr. Fusco and colleagues with great interest, and we would like to thank them for the stimulating comments regarding our paper “Medication-Related Osteonecrosis of the Jaws and CDK4/6 Inhibitors: A Recent Association”.

At the same time, we would like to take this opportunity to address their concerns and deepen some aspects that we have not been able to clearly present in our work. 

The above-mentioned case series takes into account a conceivable cumulative effect of zoledronic acid and/or denosumab during the administration of CDK inhibitors in a medication-related onset of osteonecrosis of the jaws (MRONJ).

Their position against the possible additional risk for MRONJ development eventually conferred by CDK inhibitors is mainly based on the methodological characteristics of our work.

They argued the value of a case series in the age of evidence-based medicine, with the randomized controlled trial as the standard.

We do fully agree with Dr. Fusco that no statement can be made without the appropriate study design and statistics.

A true estimate by logistic regression analysis requires a large enough number of patients, and our investigation as an observational-type study has a lot of weaknesses, as the text made it clear; thus, we agree that more studies are needed to address these unresolved questions.

Nevertheless, our work aims to provide a contribution to the developmental literature on MRONJ for at least three reasons supporting our hypothesis in this specific subset.

First, although deriving from post-marketing, Phase 1 studies, isolated data or anecdotal reports of the adverse event of osteonecrosis of the jaws have already been reported in patients receiving palbociclib [1]. A case of osteonecrosis of the jaw (1.2%) has been reported in a Phase 2 trial investigating the combination of palbociclib and letrozole [2]. Osteonecrosis of the jaw is also registered as a delayed musculoskeletal side effect for abemaciclib [3].

In these footsteps, our clinical experience does offer the opportunity for other colleagues to take the opportunity to share their valuable experience on the topic, to review their case series examining this aspect.

Second, in vitro and in vivo data [4,5] support the hypothesis that CDK play a role in osteoclast differentiation, which also affects the RANKL-mediated cell cycle arrest, leading to osteopetrosis due to an enhanced antiresorptive effect. This could be beneficial to metastatic bone disease but could eventually lead to an increased risk of MRONJ.

Third, in our report, all the patients received concomitant antiresorptive treatment; thus, of course, the association does not involve a causal relationship as it is clearly indicated. Our investigation arises from the need to define whether an unidentified trigger exists for spontaneous MRONJ, and we propose this interpretation of the possible hypothesized “association”. Our main aim is to convey the message of a possible relationship between new anti-cancer therapies and a probable co-occurrence of adversity, which should be addressed in the future since individualized MRONJ prevention strategies can only come from a thoughtful understanding of risk factors.

In this regard, our group recently conceived a case and control study to investigate the complementary rather than independent risk factors for MRONJ development.

We feel grateful and humbled for this constructive debate and look forward to a dialogue with oncologists and experts in the field to pay adequate attention to this specific subset of patients.

## Data Availability

Not applicable.
